# Definition of Personalized Medicine and Targeted Therapies: Does Medical Familiarity Matter?

**DOI:** 10.3390/jpm11010026

**Published:** 2021-01-04

**Authors:** Valentyn Fournier, Thomas Prebet, Alexandra Dormal, Maïté Brunel, Robin Cremer, Loris Schiaratura

**Affiliations:** 1 University of Lille, ULR 4072—PSITEC—Psychologie: Interactions, Temps, Emotions, Cognition, F-59000 Lille, France; maite.brunel@univ-lille.fr (M.B.); loris.schiaratura@univ-lille.fr (L.S.); 2 Yale Cancer Center, Yale University, 333 Cedar Street, New Haven, CT 06520, USA; thomas.prebet@yale.edu (T.P.); alexandra.dormal@yale.edu (A.D.); 3 Espace éthique régional des Hauts-de-France, Centre Hospitalier Universitaire de Lille, F-59037 Lille, France; robin.cremer@chru-lille.fr

**Keywords:** personalized medicine, targeted therapies, definition, attitudes, familiarity, genetics, genomics

## Abstract

Personalized medicine (PM) is increasingly becoming a topic of discussion in public health policies and media. However, there is no consensus among definitions of PM in the scientific literature and the terms used to designate it, with some definitions emphasizing patient-centered aspects and others emphasizing biomedical aspects. Furthermore, terms used to refer to PM (e.g., “pharmacogenomics” or, more often, “targeted therapies”) are diverse and differently used. To our knowledge, no study has apprehended the differences of definition and attitudes toward personalized medicine and targeted therapies according to level of familiarity with the medical field. Our cohort included 349 French students from three different academic fields, which modulated their familiarity level with the medical field. They were asked to associate words either to “personalized medicine” or “target therapies”. Then, they were asked to give an emotional valence to their associations. Results showed that nonfamiliar students perceived PM as more positive than targeted therapies (TT), whereas familiar students showed no difference. Only familiar students defined PM and TT with technical aspects such as genetics or immunology. Further studies are needed in the field in order to determine which other factors could influence the definitions of PM and TT and determine how these definitions could have an impact in a clinical setting.

## 1. Introduction

Since its rise in the early 2000s, personalized medicine (PM) has been an increasingly important topic in public health policies, often presented as a revolution in medical care in the media [1,2]. PM has followed many technical advances, notably in genetics and genomics, allowing new therapeutic options for many medical conditions such as coronary artery disease, Alzheimer’s disease, severe depression, and cancer [3,4,5]. However, the public expectations about and knowledge of PM have not grown as fast as scientific knowledge has. In addition, it seems that there is no consensus about the definition of PM [6,7,8], as two main definitions can be distinguished in the literature. These are partially overlapping and describe PM either as a patient-centered approach (i.e., adjusting treatment to the specificities of an individual patient) [9,10,11,12] or as a genomics-based treatment strategy [6,11,13].

PM seems to have a wide scope, encompassing many practices, including targeted therapies (TT) [8,10,12]. It seems there are several terms used to name PM [6,7,10,14,15]. Indeed, it is possible to define PM as “targeted therapy”, “pharmacogenomics” or “precision medicine” depending on the author, the domain or the definition [6]. This point constitutes a real issue as even if these terms are used to designate the same concept, they could possibly raise different representations or beliefs in people. Moreover, the use of different words to refer to the same concept could reinforce inaccuracies in the definition of PM.

The attitudes of people toward genetics and genomics are ambiguous, depending on the application. They are negative for some (e.g., perinatal diagnosis, genetically modified organisms or human embryo research) [15,16,17,18,19] and positive for others (i.e., when medical benefits are clearly identifiable, like in PM) [16,17,18,19]. However, given the confusion of both patients and the general population toward the understanding of genetics and genomics [16,17,18,19], unrealistic expectations toward PM can be observed [20]. For healthcare professionals, attitudes toward PM seem mixed [21]. Even if they are mostly positive toward PM, professionals can express many concerns regarding potential issues engendered by PM (or its constitutive elements) such as a lack of knowledge concerning genetics and genomics, a lack of guidelines or an inability to explain the results of somatic testing to patients. When professionals and patients are compared, perceptions regarding the benefits of genetic testing and genomic medicine are different: professionals are more aware of their lack of knowledge concerning genetics and genomics and their potential negative issues (e.g., misinformation about the results, incidental findings) [20,22]. These findings match the assumption that the more people know about genetics, the more they will have concerns toward its applications, despite their positive attitudes [1,23]. On the contrary, some authors also observed that the study field of students can modulate their attitudes toward pharmacogenomics and PM. Indeed, according to their study field, students can have more or less positive attitudes toward pharmacogenomics and PM. For example, students in health fields have more positive attitudes than nonhealth students [24]. From these results, we can assume that familiarity with the medical field and/or genetics- and genomics-related concepts has an impact on attitudes toward PM.

To our knowledge, no research has studied the specific effect of familiarity with the medical domain on the definition of PM and representations deriving from the different words used to refer to it (e.g., targeted therapies (TT)). Thus, the main objective of this research was to examine how familiarity with the medical domain could impact the definitions of PM and TT. Familiarity was manipulated by selecting students from the different study fields of English foreign language, psychology and medicine.

Even if they shared some common definitional elements, we hypothesized that the definition of PM would be different from the representation of TT (i.e., TT would have a narrower scope than PM). Regarding the global attitudinal charge of the terms, PM would be evaluated more positively than TT. More specifically, PM would be defined with words referring to more humanistic and patient-centered aspects while TT would be defined with words referring to more technical aspects.

We expected that familiarity with the medical field would influence the definition of and attitude toward PM and TT. Compared with the least familiar students, the medicine students would show more similarities in the way they define PM and TT. In more detail, we hypothesized that, in medicine students, some of the most frequent words used to define both PM and TT would be relative to technical, biological or genetics- or genomics-related aspects and that these elements would not be reflected in the other groups (English foreign language students and psychology students). In line with their familiarity with the medical field, the medicine students would also evaluate PM and TT in the same way on an attitudinal level, whereas PM would be evaluated more positively than TT by the less familiar students.

## 2. Materials and Methods

### 2.1. Overview

To study the representations of PM and TT, we used a free association task, following the recommendations of Lo Monaco et al. (2016) [25] (also see [26]). Participants were divided into two groups. After having received a very short framing text, each participant was asked to associate the five words or expressions that spontaneously came to mind when the stimuli words “personalized medicine” or “targeted therapy” were provided. The constraint of a limited number of words leads to a possibly homogenized range of verbal results from the participants [27]. Subsequently, participants were asked to attribute an attitude score (i.e., degree of attraction or aversion) to the words they cited. This measure led to the identification of the global underlying total mean emotional value of each concept in each group (for a similar method, see [28,29]).

This research received the favorable opinion of the Research Ethics Board in Behavioral Sciences of the University of Lille (ref. 2018-295-S63). According to French legislation, the research was noninterventional and conducted on a nonclinical population. The participants consented to take part in the study after being informed of the conditions and goals of the study.

### 2.2. Participants

In this study, two corpuses were created, comprising 349 students in their third year of university studies (258 females and 91 males), of which 249 had a relative who currently or previously had cancer, in a similar proportion (comprising between 69.8% and 77.4%) in all groups (see sociodemographic data in Table 1). The first corpus comprised 169 French Bachelor’s degree students (mean age = 21.10 years) in the different domains of English foreign language (*n* = 48), medicine (*n* = 59) or psychology (*n* = 62) using the stimulus words “personalized medicine” (PM). The second corpus used the stimulus words “targeted therapy” (TT) and comprised 180 French Bachelor’s degree students (mean age = 21.01 years) in English foreign language (*n* = 57), medicine (*n* = 63) or psychology (*n* = 60). Neither the difference in mean age between the “PM” group and the “TT” group (t_347_ = 0.248; *p* = 0.804) nor the difference between the age of English foreign language, medicine and psychology students (*F*_2;346_ = 0.741; *p* = 0.477) were statistically significant.

Medicine students were considered the most familiar with the medical field and genetics- and genomics-related concepts, whereas English foreign language and psychology students were considered less familiar. Indeed, the curriculum of medicine students comprised several fundamental and applied biology courses, the curriculum of psychology students did to a much lesser extent (i.e., only an introduction to biology course at the students’ level of studies) and the English foreign language curriculum had no biology courses.

### 2.3. Data Analysis

#### 2.3.1. Analysis of Attitudes toward PM and TT

First, a valence analysis was conducted in order to analyze the attitudes toward PM and TT. This analysis compared the respective emotional valence mean scores (i.e., mean of the attributed valences toward each of the words provided by participants) of PM and TT. Then, we proceeded to calculate a neutrality index (see [30]), which ranged between −1 and +1 (an index value between −1 and −0.05 meant that few words were neutrally connoted, an index value between −0.04 and +0.04 meant that the number of neutral words was equal to the number of emotionally connoted ones and an index value between +0.05 and +1 meant that most of the words were neutral).

#### 2.3.2. Definitions of PM and TT: Frequency Analysis

In order to examine the representations of PM and TT, a frequency analysis of the corpuses was conducted. The frequency of appearance of specific words or expressions was calculated. In our analysis, we focused only on the highly frequent elements (at high and low rank of appearance) which could be considered the most prototypical (i.e., cognitively accessible and shared).

The data produced by the free association task were analyzed using the IRaMuTeQ, which is an open source textual analysis software. IRaMuTeQ is used to conduct statistical analysis on textual material in terms of the appearance frequency of specific terms.

As recommended by DiGiacomo (1986) [31], we proceeded to a preliminary grouping procedure, aggregating words from the same family into the same category. Following the recommendations of Lo Monaco et al. (2016) [25], only words or expressions that were produced by more than 10% of the participants from each sample were selected.

Two types of comparisons were conducted. First, we compared the definitions of PM and the definitions of TT in the global sample. Then, we compared these two definitions between three groups of participants identified by their study domain in order to determine the role of familiarity with medical concepts in terms of attitude toward PM and TT.

## 3. Results

First, attitudes toward PM and TT will be presented, taking into account familiarity with the medical field and the interaction between familiarity level and the concept represented (i.e., PM or TT). Then, a similarity analysis between representations of PM and TT according to familiarity with the medical field will be presented. Finally, the effects of familiarity on the definitions of PM and TT will be analyzed by presenting the elements most frequently cited by participants.

### 3.1. Analysis of Attitudes toward PM and TT

All the attitude scores (rated from −2 to +2, see Table 2) were strictly positive (*p* < 0.0001). In addition, the neutrality indexes of the words were calculated. They ranged between −1 and −0.04 (see [30]). Overall, this meant that the number of neutral words was less than the number of emotionally connoted words.

Generally, the results of the analysis showed that the attitude toward PM (*M* = 0.859; *sd* = 0.613) was significantly more positive than the attitude toward TT (*M* = 0.542; *sd* = 0.697, *F*_1;341_ = 21,447; *p* < 0.001). A comparison analysis showed that this remained true for the least familiar students (i.e., English foreign language (*F*_1;103_ = 9941; *p* = 0.002) and psychology students (*F*_1;119_ = 8205; *p* = 0.005)) but not for the most familiar students (*F*_1;119_ = 3240; *p* = 0.074).

Analyses showed no interaction between familiarity and concept (i.e., PM or TT). However, following our hypothesis, post hoc tests showed that attitude toward PM was positive and did not differ between the three groups (*F*_2;165_ = 1931; *p* = 0.148). In contrast, attitude toward TT did significantly differ between the three groups (*F*_2;176_ = 7144; *p* = 0.001). The attitudes of medicine students were significantly more positive than the attitudes of English foreign language students (*p* = 0.001) and psychology students (*p* = 0.002). The scores of English foreign language students and psychology students were not significantly different (*p* = 0.871).

### 3.2. Familiarity and Representations of PM and TT

Generally, the total number of different words (including the most and the least frequent words) used to describe PM was 190 and the total number of different words used to describe TT was 142, reflecting a narrower view of TT compared to PM. However, taking familiarity into account, we observed that English foreign language students and psychology students gave a similar number of words for PM (100 and 104, respectively) and for TT (106 and 115, respectively), whereas medicine students gave more words for PM (79) than for TT (56).

In the following analysis, we focused on the most frequent words considered the most prototypical for PM or TT in each group (i.e., more frequent than the mean frequency of the words which were cited by more than 10% of the participants in each subgroup). In order to analyze the effect of familiarity on the definitions of PM and TT, we examined the most frequent elements according to concept and level of familiarity (see Table 3). Concerning PM, we observed that the notions of “individualization”, “tailoring” and “innovation” were cited by all participants. English foreign language students cited the notions of “treatment” and “listening”, and psychology students cited only “treatment”. Medicine students differed from the others by being the only group to mention “genetics” as a definitional component of PM. For TT, we observed that English foreign language students reported four elements: “individualization”, “treatment”, “illness” and “psychology”. Psychology students cited the same elements (except for “psychology”) and added “cancer”, “specific”, “care”, “target” and “listening”. As observed for PM, medicine students differed from the other students by citing medical-related items such as “immunology” and “genetics”, in addition to “innovation” and “cancer”.

## 4. Discussion

The aim of this study was to investigate the impact of familiarity with the medical field on the definitions of PM and TT, which are often used to designate the same concept.

Our hypothesis was that PM and TT would share some definitional elements but TT would be described with a narrower scope. Moreover, we hypothesized that attitudes toward PM would be more positive than attitudes toward TT because of the fact that PM would be defined with more humanistic words whereas TT would be defined with more technical and genetics- and genomics-related aspects. We also hypothesized that familiarity with the medical field would lead to more similarities between the definitions of PM and TT. In other words, PM and TT would be defined with technical words by medicine students but not other students.

Regarding the scope of PM and TT, we can observe that, in the least familiar students, PM was defined with the same total amount of words as TT. On the contrary, for the most familiar students, TT seems to have a narrower scope than PM given that fewer words were used to define it overall. This could be explained by the possibility that TT was considered a constitutive part of PM [10].

Generally, our results show that attitudes toward PM are significantly more positive than attitudes toward TT and that this difference does not arise from a greater number of neutral words in one condition. We also observed that PM was equally positively evaluated, whereas TT generated significantly more positive evaluations from the most familiar students (i.e., medicine students) than from the least familiar students (i.e., English foreign language and psychology students). Moreover, we can observe that PM and TT were identically evaluated by the most familiar students, whereas PM was more positively rated than TT by the other student groups. To understand these discrepancies in attitude toward PM and TT, we can examine their definitions according to degree of familiarity with the medical field.

Independent of the degree of familiarity with the medical field, PM was defined as an individualized, innovative and tailored practice. This is close to a person-centered approach of PM that considers it as taking into account the patient in their entirety, acknowledging their medical history and fitting their individual needs [10]. However, only the students most familiar with the medical field used the term “genetics” to define PM.

In the definition of TT, we observed that biomedical aspects (i.e., genetics and immunology) were emphasized when familiarity with the medical field was higher. Inversely, when familiarity with the medical field was lower, TT was defined with a wider scope, bringing to the fore individualization, treatment and illness.

For those most familiar with the medical field, the important presence of genetic aspects in the definition of both PM and TT could be related to a technical definition of PM [6,8]. On the other hand, the less medically educated groups’ definitions ignored genetics-related elements, focusing on the humanistic aspects of PM [10,11,12]. Thus, in line with the literature, we supposed that the particularly positive attitudes toward TT in the most familiar students derived from the “actionability” of medical concepts [32]. In other words, medical students could concretize what “genetics” or “immunology” could be used for. This observation can be linked to the fact that genetics are more positively seen if the medical benefits are clearly identified [17,18,19,33].

In medicine students, the positive attitudes toward PM and TT seemed to be in accordance with the literature on students [24]. Nevertheless, they contrasted with the observations made in the literature about more experienced health professionals. Indeed, it appears that experienced health professionals have concerns regarding PM and its applications [21]. We supposed that in the medical practice, the terrain experience can lead to a more nuanced view of PM and its results. Dedicated studies are therefore needed to examine the influence of medical practice level on attitude toward PM (for example, between more or less advanced students, specialists and general practitioners). However, our study presumed that greater familiarity with the medical field meant greater knowledge of genetics and genomics. The links are not that obvious though. Given that those fields are constantly and significantly growing, being a medical doctor does not always mean feeling literate enough, and differences in level of knowledge are observed even among physicians [21,34,35]. Thus, it seems appropriate to develop the degree of familiarity and the knowledge of medical students in order to promote communication about genetics and genomics technologies. Plus, our results show that there is a dynamic of construction in the definition of PM. In other words, lay individuals had an optimistic definition of PM, more than they had with TT, whereas medical students seemed more aware of the genetic dimension of PM.

Our study did not answer the inconsistencies extant in the literature about the links between knowledge and attitude toward genetics applications [1,16]. Indeed, the links between knowledge of genetics and attitude toward genetics depend on cultural aspects [16,36] and the types of genetics applications [37]. This reinforces the fact that, when it comes to genetics and genomics, “the existing polls indicate that negative reactions to genetic technologies might not simply reflect a lack of education or understanding” [18]. More dedicated studies are needed to clarify these links. Indeed, it has been shown that the representation of a concept could be modulated by social environment and education [38].

### 4.1. Limitations and Perspectives

Some limitations of this study were relative to the sample. There was an overrepresentation of women. In addition to this, the proportion of participants who had a relative who currently or previously had cancer was much greater than the proportion of participants who did not. Even if this variable was collected to control its effects by ensuring that proportions were identical between the groups, it could have been interesting to dispose of comparable samples (i.e., participants who had a cancer patient as a relative versus participants who did not have one) to compare data. Indeed, not having a familial history of cancer could mean that people were completely naïve about cancer treatment, whereas being a caregiver of a cancer patient could have led to the development of a lay expertise. This point should be explored through comparative studies between completely naïve people, caregivers of cancer patients and patients.

Except for familiarity with the medical field, the sample in this study was quite homogeneous for different variables (i.e., level of study and age). Nevertheless, the literature shows that there are some discrepancies in the apprehension of genetics and genomics according to sociodemographic variables [1,17,39,40]. Thus, our results are not generalizable to a patient cohort that is older and more heterogeneous. Given the implications of the misconception of PM in the clinical setting, it could be interesting to study the perception of PM in a population of patients.

Another limitation lies in the apparent lack of contextualization in the evaluation of attitudes. Indeed, the participants were asked to evaluate their attitudes toward the words they associated with the concepts of PM and TT. However, some negative words (e.g., “cancer”) were used for one concept but not for the other. In this case, the evaluation was relative to the associated word and not to the concept it was supposed to describe. In order to mitigate this limitation, further studies should ask the participants to produce a phrase on the basis of the words they cited in order to contextualize the participants’ associations [41].

### 4.2. Conclusions

To our knowledge, this is the first study to investigate a comparison between the definitions of PM and TT in different groups distinguished by level of familiarity with the medical field. We observed that these two concepts refer to two different realities and induce different attitudes. Therefore, we can conclude that familiarity with the medical field seems to have an impact on the representation of PM and TT. Future studies will have to recruit a more diverse sample of participants from both students and the general population. Moreover, for studies on a student population, the effects of education attainment could be evaluated.

Individuals may have preconceptions about PM, as they have about chemotherapy [42]. The medical communication about genetics and genomics can be complicated and represent a real health issue. This may potentially be reinforced by the eulogistic discourse of the media about PM [2]. This study points out the importance of communication about PM in the media and in medical courses.

Given the growing interest in PM in the media and scientific literature, studies about the comprehension and knowledge of PM are of great interest.

## Figures and Tables

**Table 1:** Sociodemographic data.

Concept	Group of Students	Sample Size	Gender (Females/Males)	Mean Age	Proportion of Participants with a Relative Who Currently or Previously Had Cancer
Personalized Medicine	English foreign language	48	36/12	20.02	37/48
	Psychology	62	47/15	21.62	48/62
	Medicine	59	39/20	21.79	42/59
Targeted Therapies	English foreign language	57	45/12	21.29	42/57
	Psychology	60	50/10	21.30	42/60
	Medicine	63	41/21 +1 nonbinary	20.57	44/63

**Table 2:** Mean and standard deviation of attitude score (from −2 to +2) and neutrality index for each group and concept.

Group	Concept	Mean	Standard Deviation	Neutrality Index
English foreign language	PM	0.8167	0.57142	−0.55
	TT	0.3930	0.76901	−0.572
Psychology	PM	0.7738	0.64959	−0.637
	TT	0.4133	0.73264	−0.547
Medicine	PM	0.9831	0.59803	−0.654
	TT	0.8032	0.49851	−0.516

**Table 3:** Most frequent words (with respective frequency and mean valence in brackets) used to describe personalized medicine (PM) and targeted therapy (TT) according to group.

	PM	TT
English foreign language students	Mean frequency: 11; *n* = 48	Mean frequency: 11; *n* = 57
	Individualization (24, 0.7383) Tailoring (19, 1.4737) Treatment (12, 0.75) Innovation (11, 1) Listening (11, 1.7272)	Individualization (20, 1.15) Illness (16, −1.3125) Treatment (14, 0.7857) Psychology (12, 0.4167)
Psychology students	Mean frequency: 14; *n* = 61	Mean frequency: 11; *n* = 60
	Individualization (45, 0.9778) Tailoring (29, 1.6552) Treatment (18, 0.2353) Innovation (17, 0.9412)	Care (17, 1.3529) Target (17, 0.4118) Individualization (16, 0.75) Treatment (14, 1.0714) Cancer (13, −1.2308) Listening (13, 0.9231) Illness (12, −1.6667) Specific (12, 0.4167)
Medicine students	Mean frequency: 15; *n* = 59	Mean frequency: 18; *n* = 62
	Genetics (34, 0.3529) Tailoring (30, 1.533) Individualization (27, 1.222) Innovation (16, 1)	Genetics (47, 0.4255) Cancer (36, −0.4857) Immunology (23, 0.5652) Innovation (23, 1.5217)

## Data Availability

The data presented in this study are openly available in FigShare at https://doi.org/10.6084/m9.figshare.13515188.v1.
